# Caffeinated Coffee, Decaffeinated Coffee and Endometrial Cancer Risk: A Prospective Cohort Study among US Postmenopausal Women

**DOI:** 10.3390/nu3110937

**Published:** 2011-11-02

**Authors:** Ayush Giri, Susan R. Sturgeon, Nicole Luisi, Elizabeth Bertone-Johnson, Raji Balasubramanian, Katherine W. Reeves

**Affiliations:** Division of Biostatistics and Epidemiology, Department of Public Health, School of Public Health and Health Sciences, University of Massachusetts Amherst, 715 North Pleasant Street, Amherst, MA 01003-9304, USA; Email: agiri@schoolph.umass.edu (A.G.); ssturgeon@schoolph.umass.edu (S.R.S.); nluisi@schoolph.umass.edu (N.L.); ebertone@schoolph.umass.edu (E.B.-J.); rbalasub@schoolph.umass.edu (R.B.)

**Keywords:** coffee, endometrial cancer, estrogen, insulin, body mass index

## Abstract

There is plausible biological evidence as well as epidemiologic evidence to suggest coffee consumption may lower endometrial cancer risk. We evaluated the associations between self-reported total coffee, caffeinated coffee and decaffeinated coffee, and endometrial cancer risk using the Women’s Health Initiative Observational Study Research Materials obtained from the National Heart, Lung, and Blood Institute Biological Specimen and Data Repository Coordinating Center. Our primary analyses included 45,696 women and 427 incident endometrial cancer cases, diagnosed over a total of 342,927 person-years of follow-up. We used Cox-proportional hazard models to evaluate coffee consumption and endometrial cancer risk. Overall, we did not find an association between coffee consumption and endometrial cancer risk. Compared to non-daily drinkers (none or <1 cup/day), the multivariable adjusted hazard ratios for women who drank ≥4 cups/day were 0.86 (95% confidence interval (CI) 0.63, 1.18) for total coffee, 0.89 (95% CI 0.63, 1.27) for caffeinated coffee, and 0.51 (95% CI 0.25, 1.03) for decaf coffee. In subgroup analyses by body mass index (BMI) there were no associations among normal-weight and overweight women for total coffee and caffeinated coffee. However among obese women, compared to the referent group (none or <1 cup/day), the hazard ratios for women who drank ≥2 cups/day were: 0.72 (95% CI 0.50, 1.04) for total coffee and 0.66 (95% CI 0.45, 0.97) for caffeinated coffee. Hazard ratios for women who drank ≥2 cups/day for decaffeinated coffee drinkers were 0.67 (0.43-1.06), 0.93 (0.55-1.58) and 0.80 (0.49-1.30) for normal, overweight and obese women, respectively. Our study suggests that caffeinated coffee consumption may be associated with lower endometrial cancer risk among obese postmenopausal women, but the association with decaffeinated coffee remains unclear.

## 1. Introduction

Known risk factors for endometrial cancer, such as use of unopposed estrogen, obesity, early menarche, and late age at menopause, support a hormonal etiology [1,2,3,4,5,6]. In support of this etiology, decreased plasma sex hormone binding globulin (SHBG) levels and elevated estrogen levels are associated with increased endometrial cancer risk [7,8]. Further, increased insulin resistance, hyperinsulinemia, and diabetes may increase endometrial cancer risk [1,2,9]. Coffee and caffeine consumption have been associated with increased plasma sex hormone binding globulin (SHBG) levels, decreased free estradiol levels [10,11,12,13], and improved insulin sensitivity [14]. Thus, it is biologically plausible that coffee consumption may reduce endometrial cancer risk. Additionally, since obese post-menopausal women have higher circulating estrogen [6] and insulin levels [15] than thinner post-menopausal women, the effect of coffee in reducing endometrial cancer risk may be greatest among these women. Although the association between obesity and endometrial cancer risk has been established, evidence linking dietary factors and endometrial cancer risk are limited [16].

To date three prospective cohort studies have evaluated the association between coffee consumption and endometrial cancer risk [17,18,19]. The only prospective study to examine this relationship by body mass index (BMI) subgroups observed the greatest reduction in risk among obese women [17]. Additionally, to our knowledge, no prospective study has examined the independent associations between caffeinated and decaffeinated coffee and endometrial cancer risk.

We utilized the Women’s Health Initiative (WHI) Observational Study (OS) Research Materials obtained from the National Heart, Lung, and Blood Institute (NHLBI) Biological Specimen and Data Repository Coordinating Center to prospectively examine the association between coffee and endometrial cancer risk. With a large study population and access to detailed information on coffee consumption, these data provided a unique opportunity for evaluating the independent associations of caffeinated coffee and decaffeinated coffee overall and by BMI status.

## 2. Methods

### 2.1. Study Population

The WHI OS is a multi-center, ethnically diverse prospective cohort study that enrolled 93,676 eligible women aged 50-79 years. Details regarding study design, participant enrollment and eligibility have been previously described [20]. Briefly, post-menopausal women who were unwilling or ineligible to participate in the clinical trial arms of the WHI study, did not plan to move and/or had expected survival time of three or more years were eligible to participate in the study. Participants were recruited at 40 clinical centers throughout the US between 1 October 1993 and 21 December 1998. We accessed publicly available data from the WHI OS with follow-up through 12 September 2005.

### 2.2. Exclusion Criteria

From the 93,676 women at baseline, we excluded participants from analysis who reported having a previous cancer other than non-melanoma skin cancer (*n* = 5355), hysterectomy (*n* = 32,429) or both-previous cancer other than non-melanoma skin cancer and hysterectomy (*n* = 6720) at baseline. We additionally excluded participants for whom there was no information available on follow-up (*n* = 260) or coffee consumption (*n* = 1935). Our primary analyses included 45,696 participants who contributed a total of 342,927 total person-years of follow-up. The average follow-up among the 45,696 participants was 7.5 years. 

### 2.3. Exposure Variables

Data on coffee consumption were collected at baseline using a structured questionnaire. Participants were asked “Do you usually drink coffee each day?” Participants answering “Yes” further indicated separately the number of cups of caffeinated and decaffeinated coffee they drank each day: None, 1 cup/day, 2-3 cups/day, 4-5 cups/day, ≥6 cups/day. We created three coffee consumption variables: cups of total coffee consumed per day (caffeinated and/or decaffeinated coffee combined), cups of caffeinated coffee each day and cups of decaffeinated coffee each day. Participants who did not drink coffee on a daily basis served as the referent category for all analyses. Women also reported coffee consumption on a similarly worded questionnaire administered at year 3 of follow-up.

### 2.4. Case Determination

Adjudication of endometrial cancer cases in the WHI has been described previously [21]. Briefly, endometrial cancer cases were initially self-reported through annual questionnaires or at clinic visits every three years. Cases were locally and centrally adjudicated by trained physicians. Among our study population, 452 incident endometrial cancer cases were adjudicated during follow-up, among which 427 cases had complete information on key covariates.

### 2.5. Statistical Analysis

Baseline characteristics of participants were compared by cups of total coffee, caffeinated coffee and decaffeinated coffee consumption categories using chi-square tests or ANOVA as applicable. All coffee variables were categorized as: none or <1 cup/day (referent category), 1 cup/day, 2-3 cups/day and ≥4 cups/day for the overall analyses, and none or <1 cup/day, 1 cup/day and ≥2 cups/day for subgroup analyses, due to small numbers. Cox proportional hazard (PH) regression models were used to estimate hazard ratios (HR) and 95% confidence intervals (95% CI) for total coffee, caffeinated coffee, and decaffeinated coffee consumption. We considered as potential confounders factors associated with coffee consumption as well as known or suspected risk factors for endometrial cancer. Variables initially considered were: age (years; continuous), ethnicity (white, black and other), unopposed estrogen use (never, past, current), combined estrogen-progestin use (never, past, current), smoking (never, past, current), BMI (kg/m^2^; continuous), age at menarche (<12, 12-13, >13 years), age at menopause (years; continuous), parity (No full term pregnancy, 1-2 full term pregnancies, ≥3 full term pregnancies), tea consumption (0, 1, 2-3, ≥4 cups/day), diabetes status (never, ever), physical activity (metabolic equivalents (METs/week; continuous)), history of oral contraceptive use (never, ever), family history of endometrial cancer (yes, no, don’t know) and total daily energy intake (kcal; continuous). We created a parsimonious model using stepwise selection and likelihood ratio tests, and adjusted for age, ethnicity, unopposed estrogen use, combined estrogen-progestin use, smoking, and BMI. Given its strong association with coffee consumption, we separately adjusted for cigarette smoking by duration of regular smoking (never/<100 cigarettes during lifetime, ≤9 years, 10-29 years, ≥30 years), and pack-years of smoking (0, 1-9, 10-39, ≥40), and similar results were obtained using each approach. In models assessing caffeinated coffee and decaffeinated coffee, types of coffee were mutually adjusted for each other. Simultaneously adjusting for all other considered variables did not change our findings. Follow-up time for each participant was accrued from enrollment to date of diagnosis of endometrial cancer, date of hysterectomy, death, or date of last follow-up contact, whichever came first. Women who had a diagnosis of any other cancer during follow-up were not censored and were considered to be at risk of endometrial cancer. The assumption of proportional hazards in the Cox PH models was tested by including an interaction term with time, for all beverage consumption variables individually. There was no significant evidence of violation of the PH assumption in all reported models.

We conducted further sub-group analyses by BMI as reported at baseline (<25 kg/m^2^, 25-29.9 kg/m^2^, and ≥30 kg/m^2^) adjusted for age only and for variables reported in the parsimonious multivariable adjusted models, for all coffee variables. For the sub-group analyses we only report results from the parsimonious models, as age adjusted hazard ratios were not different. We additionally performed analyses restricted to participants that reported drinking exclusively caffeinated coffee or drinking decaffeinated coffee at baseline. In the overall analyses, we formally tested for interaction between BMI and coffee consumption by adding an interaction term to our multivariable regression models.

We conducted sensitivity analyses restricting the study population to those participants who were concordant in reporting their coffee consumption category at baseline and year 3 of follow-up. For these analyses, individuals who developed endometrial cancer, or had a hysterectomy, or had a censoring event (death, date of last-follow up contact) prior to the year 3 time point were not included.

Two-sided *p* values <0.05 were considered statistically significant, with no adjustment for multiple comparisons. All analyses were performed using SAS version 9.2 (Cary, NC).

## 3. Results

The majority of women (71.1%) reported drinking coffee (caffeinated or decaffeinated) on a daily basis at baseline. Of these daily coffee consumers, the proportion of women that drank caffeinated coffee only, decaffeinated coffee only, and both was 53.4%, 19.3%, and 27.3%, respectively. Compared to non-daily drinkers, total coffee consumers were more likely to be white and current smokers, and less likely to be obese and non-smokers (Table 1). Similar patterns were seen for both caffeinated coffee only consumers and decaffeinated coffee only consumers (data not shown). Daily coffee consumers were also significantly less likely to have diabetes at baseline (Table 1), however this trend was only in caffeinated coffee consumers and not in decaffeinated coffee consumers (data not shown). 

**Table 1:** Characteristics of study participants at baseline according to coffee consumption in the Women’s Health Initiative Observational Study (*n* = 46,977).

	Daily total coffee consumption							
	0 or <1 cup/day		1 cup/day		2-3 cups/day		≥4 cups/day	
**Continuous**	**Mean**	**(SD)**	**Mean**	**(SD)**	**Mean**	**(SD)**	**Mean**	**(SD)**
Age (years)	62.7	(7.5)	64.0	(7.4)	63.3	(7.2)	62.9	(7.1)
Physical exercise (MET/week)	14.6	(15.0)	13.7	(14.4)	14.5	(14.3)	14.3	(14.7)
Age at menopause	50.3	(4.9)	50.3	(5.1)	50.3	(5.1)	50.0	(4.8)
Body mass index	27.1	(6.1)	26.8	(5.6)	26.8	(5.4)	26.8	(5.3)
Total daily energy intake (kcal)	1528	(677)	1501	(689)	1554	(650)	1642	(716)
**Categorical**	***n*^†^**	**%**	***n*^†^**	**%**	***n*^†^**	**%**	***n*^†^**	**%**
Ethnicity								
White	10,828	(80.1)	5347	(78.6)	16,737	(88.5)	7071	(93.2)
Black	1413	(10.5)	608	(8.9)	754	(4.0)	141	(1.9)
Other	1284	(9.5)	851	(12.5)	1428	(7.6)	374	(4.9)
Body mass index								
Normal BMI (<25 kg/m^2^)	5986	(44.6)	3028	(44.9)	8183	(43.6)	3177	(42.1)
Overweight (25-30 kg/m^2^)	4110	(30.6)	2186	(32.4)	6420	(34.2)	2711	(36.0)
Obese (≥30 kg/m^2^)	3327	(24.8)	1533	(22.7)	4157	(22.2)	1651	(21.9)
Age at menarche								
Early menarche (<12 years)	2822	(20.9)	1347	(19.8)	4044	(21.4)	1682	(22.2)
Average (12-13 years)	7395	(57.7)	3746	(55.1)	10,578	(56.0)	4190	(55.4)
Late menarche (>13 years)	3301	(24.4)	1710	(25.1)	4272	(22.6)	1694	(22.4)
Number of full term births								
None	2044	(15.2)	881	(13.0)	2468	(13.1)	1014	(13.4)
1-2	4891	(36.3)	2464	(36.3)	6586	(35.0)	2620	(34.7)
3 or more	6541	(48.5)	3439	(50.7)	9782	(51.9)	3911	(51.8)
Smoking status								
Never smoked	8243	(61.4)	3752	(55.7)	8656	(46.2)	2855	(37.1)
Past smoker	4741	(35.3)	2698	(40.1)	8907	(47.6)	3664	(48.9)
Current smoker	444	(3.3)	282	(4.2)	1157	(6.2)	981	(13.1)
Oral contraceptive use								
Never	7859	(57.9)	4187	(61.3)	11,038	(58.2)	4430	(58.3)
Ever	5712	(42.1)	2643	(38.7)	7935	(41.8)	3173	(41.7)
Diabetes Status								
No	12,581	(94.8)	6457	(94.6)	18,128	(95.7)	7289	(95.9)
Yes	705	(5.2)	370	(5.4)	824	(4.3)	310	(4.1)
Combined estrogen-progestin use								
Never used	7917	(58.4)	3920	(57.4)	10,527	(55.5)	4385	(57.7)
Past user	1173	(8.7)	615	(9.0)	1657	(8.7)	653	(8.6)
Current user	4472	(33.0)	2290	(33.6)	6779	(35.8)	2562	(33.7)
Unopposed estrogen use ^‡^								
Never used	11,957	(88.1)	5956	(87.3)	16,652	(87.8)	6722	(88.5)
Past user	1242	(9.2)	680	(10.0)	1832	(9.7)	698	(9.2)
Current user	368	(2.7)	190	(2.8)	482	(2.5)	179	(2.4)
Family history of endometrial cancer ^‡^								
No	221	(1.6)	89	(1.3)	238	(1.3)	103	(1.4)
Yes	5670	(41.8)	2947	(43.2)	8274	(43.6)	3229	(42.5)
Don’t know	7680	(56.6)	3794	(55.5)	10,461	(55.1)	4271	(56.1)

^†^ Numbers may not add to 46,977 because of missing values. Due to the large sample size *p* values for all variables were <0.0001, except for ^‡^ unopposed estrogen use (*p* = 0.19); and ^‡^ family history of endometrial cancer (*p* = 0.96).

We observed no overall inverse associations for total coffee, and caffeinated coffee and a marginally statistically significant inverse association between decaffeinated coffee and endometrial cancer risk. Compared to non-drinkers, the multivariable adjusted hazard ratios (HR) and 95% confidence intervals (95% CI) for women who drank ≥4 cups were: 0.86 (95% CI: 0.63-1.18) for total coffee, 0.89 (0.63-1.27) for caffeinated coffee only, and 0.51 (0.25-1.03) for decaffeinated coffee only (Table 2).

In sub-group analyses by BMI category, there were no statistically significant associations among normal weight and overweight women for total coffee, or caffeinated coffee consumption with endometrial cancer risk (Table 3). For decaffeinated coffee consumption there was a marginally significant association among normal weight women who drank ≥2 cups of decaffeinated coffee each day (HR: 0.67 (95% CI: 0.43-1.06)). However, this association among normal weight women was less apparent when we limited our analysis to women who exclusively drank decaffeinated coffee only (*n* = 8834); HR: 0.88 (95% CI: 0.57-1.57). We did not find any association among overweight women who drank ≥2 cups of decaffeinated coffee each day (HR: 0.93 (0.55-1.58)).

In obese women, there was a borderline-significant lower risk (HR 0.72 (95% CI 0.50-1.04)) among women who drank ≥2 cups of total coffee/day, compared to obese women who were non-daily drinkers (Table 3). This inverse association among obese women was stronger and was statistically significant among women who drank ≥2 cups of caffeinated coffee/day (HR: 0.66 (95% CI: 0.45-0.97)) but less pronounced among women who drank ≥2 cups of decaffeinated coffee/each day (HR: 0.80 (95% CI: 0.49-1.30)) (Table 3). Restricting our analyses to obese women who exclusively drank either caffeinated coffee only (*n* = 7511) or decaffeinated drinkers only (*n* = 4649), strengthened the effect estimates for both caffeinated coffee (HR: 0.60 (95% CI: 0.39-0.93)) and decaffeinated coffee (HR: 0.70 (0.37-1.33)). The interactions between the three coffee variables and BMI were not statistically significant; with p values of 0.38, 0.30 and 0.51 for total coffee, caffeinated coffee and decaffeinated coffee, respectively.

**Table 2:** Multivariable adjusted hazard ratios (HRs) and 95% confidence intervals (CIs) of total coffee, caffeinated coffee and decaffeinated coffee in relation to endometrial cancer in the Women’s Health Initiative Observational Study (*N* = 45,696).

Beverage consumption	0 or <1 cup/day	1 cup/day	2-3 cups/day	≥4 cups/day	*p*-val ^a^ (trend) ^b^
Total Coffee					
Incident cases	126	71	168	62	
Person-years	98,679	49,010	138,726	56,512	
Age adjusted HR (95%CI) ^†^	1.00 (ref)	1.07 (0.80-1.43)	0.92 (0.73-1.50)	0.85 (0.63-1.15)	
Multivariable adjusted HR (95% CI) ^‡^	1.00 (ref)	1.12 (0.84-1.50)	0.91 (0.72-1.16)	0.86 (0.63-1.18)	0.41 (0.23)
**Caffeinated Coffee**					
Incident cases	185	81	121	40	
Person-years	148,060	57,188	101,564	36,112	
Age adjusted HR (95% CI) ^†^	1.00 (ref)	1.13 (0.82-1.57)	0.89 (0.68-1.17)	0.96 (0.67-1.39)	
Multivariable adjusted HR (95% CI) ^‡^	1.00 (ref)	1.15 (0.86-1.45)	0.93 (0.73-1.17)	0.89 (0.63-1.27)	0.57 (0.45)
**Decaffeinated Coffee**					
Incident cases	299	68	52	8	
Person-years	234,645	51,397	45,428	11,454	
Age adjusted HR (95% CI) ^†^	1.00 (ref)	1.01 (0.78-1.32)	0.88 (0.65-1.18)	0.54 (0.27-1.09)	
Multivariable adjusted HR (95% CI) ^‡^	1.00 (ref)	0.99 (0.76-1.30)	0.84 (0.63-1.13)	0.51 (0.25-1.03)	0.19 (0.07)

^†^ Age adjusted hazard ratios (452 cases); ^‡^ Multivariable adjusted hazard ratios from Cox-proportional hazard regression models: adjusted for age (continuous), ethnicity (white, black and other), unopposed estrogen use (never, past, current), progestin + estrogen use (never, past, current), smoking (never, past, current) and BMI (continuous). When we examined caffeinated coffee or decaffeinated coffee in relation to endometrial cancer risk, each beverage was mutually adjusted for each other; ^a^ Represents *p*-value from likelihood ratio test for categorical coffee variables; ^b^ Represents *p*-value from linear trend test across coffee categories.

**Table 3:** Multivariable HRs and 95% CIs of total coffee, caffeinated coffee and decaffeinated coffee consumption in relation to endometrial cancer by body mass index status in the Women’s Health Initiative Observational Study (*N* = 45,696).

Beverage consumption	0 or <1 cup/day	1 cup/day	≥2 cups/day	*p*-val ^a^ (trend) ^b^	*P*-int ^c^
**Total Coffee**					**0.38**
**Normal-weight (BMI < 25 kg/m^2^)**					
** *N* = 20,039**					
Incident cases	50	27	99		
Person-years	44,601	22,450	84,986		
HR (95% CI) ^‡^	1.00 (ref)	1.03 (0.64-1.64)	1.02 (0.72-1.45)	0.99 (0.90)	
**Overweight (BMI 25 kg/m^2^-29.9 kg/m^2^) **					
** *N* = 15,159**					
Incident cases	26	17	60		
Person-years	30,127	15,619	67,741		
HR (95% CI) ^‡^	1.00 (ref)	1.21 (0.66-2.32)	1.01 (0.63-1.60)	0.79 (0.93)	
**Obese (BMI ≥ 30 kg/m^2^)**					
** *N* = 10,498**					
Incident cases	50	27	71		
Person-years	23,951	10,939	42,511		
HR (95% CI) ^‡^	1.00 (ref)	1.20 (0.70-1.79)	0.72 (0.50-1.04)	0.08 (0.06)	
**Caffeinated Coffee**					**0.3**
**Normal-weight (BMI < 25 kg/m^2^)**					
** *N* = 20,039**					
Incident cases	70	34	72		
Person-years	66,860	26,212	58,965		
HR (95% CI) ^‡^	1.00 (ref)	1.23 (0.81-1.87)	1.17 (0.83-1.64)	0.53 (0.37)	
**Overweight (BMI 25 kg/m^2^-29.9 kg/m^2^)**					
** *N* = 15,159**					
Incident cases	44	16	43		
Person-years	46,985	18,717	47,785		
HR (95% CI) ^‡^	1.00 (ref)	0.88 (0.50-1.58)	0.99 (0.65-1.53)	0.91 (0.99)	
**Obese (BMI ≥ 30 kg/m^2^)**					
** *N* = 10,498**					
Incident cases	71	31	46		
Person-years	34,215	12,259	30,927		
HR (95% CI) ^‡^	1.00 (ref)	1.16 (0.75-1.78)	0.66 (0.45-0.97)	0.03 (0.05)	
**Decaffeinated Coffee**					**0.51**
**Normal-weight (BMI < 25 kg/m^2^)**					
** *N* = 20,039**					
Incident cases	129	25	22		
Person-years	102,756	23,973	25,307		
HR (95% CI) ^‡^	1.00 (ref)	0.77 (0.50-1.18)	0.67 (0.43-1.06)	0.15 (0.06)	
**Overweight (BMI 25 kg/m^2^-29.9 kg/m^2^)**					
** *N* = 15,159**					
Incident cases	68	17	18		
Person-years	76,691	16,583	20,212		
HR (95% CI) ^‡^	1.00 (ref)	1.10 (0.64-1.87)	0.93 (0.55-1.58)	0.90 (0.86)	
**Obese (BMI ≥ 30 kg/m^2^)**					
** *N* = 10,498**					
Incident cases	102	26	20		
Person-years	55,198	10,840	11,362		
HR (95% CI) ^‡^	1.00 (ref)	1.17 (0.76-1.82)	0.80 (0.49-1.30)		

^‡^ Multivariable adjusted hazard ratios from Cox-proportional hazard regression models: adjusted for age (continuous), ethnicity (white, black and other), unopposed estrogen use (never, past, current), combined estrogen-progestin use (never, past, current), and smoking (never, past, current). When we examined caffeinated coffee or decaffeinated coffee in relation to endometrial cancer risk, each beverage was mutually adjusted for each other; ^a^ Represents *p*-value from likelihood ratio test for categorical coffee variables; ^b^ Represents *p*-value from linear trend test across coffee categories; ^c^ Represents *p*-value for interaction.

Adjustment for cigarette smoking by duration of smoking or pack-years of smoking separately while adjusting for variables included in the main models did not change our results. After adjusting for duration of smoking, the multivariable adjusted HRs and 95% CI among obese women who drank ≥2 cups of caffeinated coffee and decaffeinated coffee were 0.65 (0.44-0.95) and 0.79 (0.49-1.29) respectively, compared to non-daily drinkers. Similarly, after adjusting for pack-years of smoking associations were 0.66 (0.45-0.97) and 0.78 (0.47-1.27) for caffeinated coffee and decaffeinated coffee, respectively.

We observed similar results in analyses further restricted to women who reported drinking the same number of coffee cups/day category at both baseline and year 3 (*n* = 3898). However, the inverse association between ≥2 cups caffeinated coffee consumption and endometrial cancer risk among obese women was strengthened (HR: 0.40 (95% CI: 0.18-0.91)).

We also examined the association between tea consumption and endometrial cancer risk, while adjusting for variables in the parsimonious models and coffee consumption. No statistically significant associations were observed overall (multivariable HR 1.10, 95% CI 0.61-1.97 for ≥4 cups/day *vs.* non-daily tea consumption) or within BMI categories (data not shown).

## 4. Discussion

In this large prospective cohort study of post-menopausal women, we found some evidence of an overall inverse association between decaffeinated coffee and endometrial cancer risk, and no such overall associations for total coffee, or caffeinated coffee. However, within the subgroup of obese women (baseline BMI ≥ 30 kg/m^2^), we observed a lower risk of endometrial cancer among obese women who drank ≥2 cups/day of coffee. Though these observations were based on a small number of cases, the results suggest that coffee consumption may lower endometrial cancer risk among obese women, who also have the highest absolute risk in comparison to thin women.

Three other studies [17,18,19] have prospectively evaluated coffee consumption and endometrial cancer risk. The Swedish Mammography Cohort, the large study of predominantly post-menopausal women who primarily drank caffeinated coffee, reported an overall 25% lower risk in the highest category of coffee drinkers [17]. Additionally, they showed that the reduction in risk was strongest among heavy women and not present in normal weight women. Our results are most consistent with this Swedish study as we too found an inverse association between caffeinated coffee consumption and endometrial cancer risk among obese women [17]. A second prospective study conducted in Japan reported a 62% overall lower risk among women with the highest coffee consumption [18]. The smallest prospective study in Norway did not show an inverse association between coffee intake and endometrial cancer risk [19]. However, the referent category in this study included women who drank ≤2 cups of coffee per day and over 60% of the women were pre-menopausal at baseline. Furthermore, neither of the two studies from Japan or Norway evaluated risk by BMI strata. To our knowledge our study is the first to prospectively evaluate decaffeinated coffee and endometrial cancer risk. Our results were slightly suggestive of an inverse association, with heterogeneous results across BMI strata. Only two case-control studies have evaluated decaffeinated coffee and both showed null associations [22,23].

The unopposed estrogen hypothesis provides a compelling explanation for the etiology of most endometrial cancer [5]. It posits that estrogen exposure stimulates uncontrolled cell division in the endometrium, increasing the likelihood of deleterious mutations that may result in endometrial cancer [24]. Consistent with this hypothesis, studies indicate postmenopausal women with higher serum estrogens have an increased risk of endometrial cancer [7,8]. In addition, lower blood levels of SHBG are also associated with increased endometrial cancer risk [7,8], presumably because SHBG binds to estradiol, thereby reducing the levels of bio-available estrogen. Diabetes and hyperinsulinemia are also hypothesized to play a role in endometrial carcinogenesis [1,2,9]. Specifically, insulin may promote endometrial cancer development directly as a mitogen [25] or indirectly through its effects on estrogen availability [26,27]. Several biological mechanisms related to these two pathways could account for a possible inverse association between caffeinated coffee intake and endometrial cancer risk. In line with the well-established estrogen-mediated etiology of endometrial cancer, higher intake of caffeinated coffee or caffeine has been associated with increased blood levels of sex hormone binding globulin in postmenopausal women [10,11,12,13,28]. In postmenopausal women, caffeinated coffee intake has also been associated with blood markers of decreased insulin secretion and improved insulin sensitivity, including lower C-peptide levels [29], and higher adiponectin levels [30]. In a study by Wu and colleagues, the inverse association between caffeinated coffee consumption and plasma C-peptide levels was stronger in obese and overweight women than in normal-weight women [30]. This observation is consistent with our findings and those of Friberg and colleagues who showed that the reduction in risk through caffeinated coffee consumption was greatest among heavier women [17]. Coffee also has a number of different phytochemicals other than caffeine that could also play a role in endometrial cancer carcinogenesis. For example, chlorogenic acid has been suggested to also delay glucose absorption and improve insulin sensitivity [31,32,33]. Other constituents of coffee may act as antioxidants [34] or have anti-estrogenic properties [35,36] that could also have relevance to endometrial carcinogenesis, particularly in heavier women.

We observed an overall association with decaffeinated coffee, although the pattern was not consistently observed across all body mass index categories. Decaffeinated coffee intake has been linked to decreased C-peptide levels in postmenopausal women [29]. Contrary to observations for caffeinated coffee intake, however, decaffeinated coffee intake has not been linked to elevated blood levels of adiponectin [30], elevated levels of SHBG [28], or lower levels of total estrogens [28]. A possible explanation for inverse association for decaffeinated coffee we observed is chance, as only a small percentage of our population drank decaffeinated coffee daily. Alternatively, our findings for decaffeinated coffee may reflect confounding by unknown factors, as decaffeinated coffee drinkers may be a unique population. The results for decaffeinated coffee are unclear and additional large studies that are able to separate caffeinated and de-caffeinated coffee may shed further light on this issue.

Major strengths of our study include its prospective design, detailed information on coffee consumption and potential covariates, and adjudication of endometrial cancer cases. Due to the prospective nature of the study and limited loss to follow up, the likelihood of recall bias and selection bias are minimal. Additionally, the large study population allowed us to examine the independent effects of caffeinated and decaffeinated coffee consumption. Our study is limited by its reliance on self-reported values for coffee consumption without specification of serving size per cup, which may have attenuated our results to towards the null. We additionally did not have information on other sources of caffeine, such as soft drinks and energy drinks, nor were data available on use of additives such as sugar, honey, and cream. Furthermore, we did not have access to DNA samples and were not able to evaluate potential gene-diet interactions. For example, polymorphisms of *CYP1A2* gene are associated with differential metabolism of caffeine [37] and estrogen [38]. Examination of such gene-diet interactions in future studies may help to elucidate the underlying mechanisms associated with coffee consumption and endometrial cancer risk. 

## 5. Conclusions

In conclusion, we extend evidence from previous publications in suggesting a lower risk of endometrial cancer among obese women for caffeinated coffee. Our results for decaffeinated coffee consumption and endometrial cancer risk were slightly suggestive of an inverse association; however we observed an inconsistent pattern in subgroups of women defined by body mass index. Furthermore, our study adds to an increasing body of evidence that suggests an inverse association between coffee consumption and several types of cancer including, bladder, breast, endometrial, hepatocellular, pancreatic and prostate cancer [39].
